# Plasma levels of some coagulation parameters in Steady State HBSC disease patients

**DOI:** 10.11604/pamj.2014.19.289.4451

**Published:** 2014-11-17

**Authors:** Mauryne Debola Ajuwon, Edeghonghon Olayemi, Amma Anima Benneh

**Affiliations:** 1 Department of Medical Laboratory Technology, School of Allied Health Sciences University of Ghana, Accra, Ghana; 2 Ghana Institute of Clinical Genetics and Department of Haematology, University of Ghana, Accra, Ghana; 3 Department of Haematology, University of Ghana, Accra, Ghana

**Keywords:** Coagulation parameters, steady State, HbSC disease, Ghana

## Abstract

**Introduction:**

Sickle cell disease is a collection of autosomal recessive genetic disorders. It includes homozygous HbSS and double heterozygote combinations such as HbSC. Central and West Africa bears a significant burden of HbSC disease.

**Methods:**

Prothrombin time (PT), activated partial thromboplastin time (APTT), fibrinogen concentration (FC) and platelet count (PC) were determined in 41HbSC and HbSS patients in steady state along with 40 apparently healthy HbAA controls. One way ANOVA test was used to compare means; p values< 0.05 were considered statistically significant.

**Results:**

There was no significant difference in mean PT for the study groups (p = 0.192). Mean PC was highest in HbSS patients: 445.7 +/- 128.3 X 10 9/ L compared to HbSC: 330.0 +/- 97.7 X 10 9/ L andHbAA:245 +/- 77.7 X 10 9/ L (p= 0.000). Mean APTT was 28.1 +/- 3.8 seconds in controls,24.1 + /- 66 seconds in HbSS patients and 21.8 +/- 3.8 seconds in HbSC patients (p = 0.000). Mean FC in HbSS was 1.6 +/- 0.7 g/L, 3.2 +/- 0.6 g/L in HbSC and 2.9 +/- 0.4 g/L in HbAA (p =0.000).

**Conclusion:**

A significant difference exists in PC, APTT and FC in HbSC patients compared to HbSS patients and HbAA controls. Elevated FC and shortened APTT may play a role in complications more characteristic of HbSC such as retinopathy and osteonecrosis. These suggestHbSC is not merely a milder form of HbSS; both diseases should be seen as different entities with regards to approaches for management.

## Introduction

Sickle cell disease (SCD) refers to a collection of autosomal recessive genetic disorders characterized by the presence of haemoglobin S (Hb S). This results in the clinical manifestations of sickling. Haemoglobin S is the result of a single nucleotide mutation which causes substitution of valine for glutamic acid at the sixth position from the N-terminus of the beta globin chain. Sickle cell disease includes homozygous HbSS (sickle cell anaemia), and double heterozygote combinations such as HbSC (in HbC, lysine replaces glutamic acid at position 6 from the N-terminus in the beta globin chain). The presence of Hb S is a major genetic disorder in tropical Africa with a heterozygote frequency of about 20-40% [1]. In Ghana, it has been reported that 2-3% of children are born with SCD annually [2].

It is estimated that globally almost 55,000 children are born annually with HbSC disease; with Central and West African bearing a significant burden of the disease [3, 4]. HbC is believed to offer some protection against malaria [5].

In HbSC disease, Hb C enhances the pathogenic properties of Hb S. Though HbSC and HbSS share common clinical features and complications, these were said to be milder, less frequent and to occur much later in life in HbSC compared to HbSS patients [6, 7]. As a result, HbSC disease has long been considered to be a milder variant of HbSS disease; but a study by Lamarre et al in 2012 suggests that it is a distinct disease entity [8], with a greater prevalence of retinopathy, sensori-neural disorders and osteonecrosis than HbSS. This has been attributed to the greater blood viscosity observed in HbSC patients [9].

It is also known that the variation in the frequency and severity of symptoms and complications in individuals who have SCD is only partially explained by genetic and environmental factors [10]. While there is no doubt that SCD patients have a hypercoagulable state [11], most studies have been done in HbSS patients; the objective of this study is to document plasma levels of specific coagulation parameters in HbSC patients comparing them to levels in HbSS patients and HbAA controls.

## Methods

The study population was made up of 41 previously diagnosed HbSC patients(23 males, 18 females) in steady state attending the outpatient sickle cell disease clinic at the Ghana Institute of Clinical Genetics. For purposes of comparison 40 apparently healthy HbAA (20 males and 20 females) students of the School of Allied Health Sciences, University of Ghana and 41 HbSS patients(21 males, 20females) in steady state from the same clinic were also recruited. Children less than 13 years old, pregnant women and patients with other forms of SCD were excluded from the study.

For this study, steady state was defined as the absence of any clinically detectable complication of SCD in the previous 8 weeks while the patient was not on any other drug other than routine drugs used in SCD patients such as folic acid [12]. The study was approved by the ethical review committee of the School of Allied Health Sciences, University of Ghana. Informed consent was also obtained from the patients or their guardians (for minors).

Five milliltres of blood was collected aseptically with minimal stasis from each study participant; 1.8 ml was put into a tri-sodium citrate tube for clotting profile tests and 3ml transferred into EDTA tubes for platelet count. Citrated samples were spurn at 1500 rpm for 15 minutes the plasma was then transported into plain tubes.

Prothrombin time (PT), activated partial thromboplastin time (APTT) and thrombin time (TT) were determined using Hemostat thromboplastin reagent, Novi-Clot Quick reagent and Bovine Thrombin reagent respectively following manufacturer's instructions. The thrombin time was used to calculate the fibrinogen concentration via Clauss method as previously described [13]. A BEHNK Electronic CM4 Coagulometer was used for all the clotting assays. The platelet count was done using a SYSMEX XT-2000i haematology analyzer.

Data obtained from the study were entered into a spreadsheet; statistical analysis was done using StatView for Windows, Version 5.0.1. The one way ANOVA test was used to compare means. A p value less than 0.05 was considered statistically significant.

## Results

The mean age for HbSC patients was 29.7 +/ - 12.9 years compared with 25.6 +/- 5.0 years for HbAA controls and 24.0 +/- 9.2 years for HbSS.

There was no significant difference in the mean PT for the study groups (p = 0.192) it was 12.9 +/- 1.2 seconds for controls, HbSS: 13.4 + /- 1.4 seconds and HbSC: 13.1 +/- 1.1 seconds. On the other hand, there was a significant difference in the platelet count for the study population as shown in Table 1: mean and standard deviation of coagulation parameters 1. Mean platelet count was 245 + /- 77.7 X 10 9/ L for controls, 445.7 + /- 128.3 X 10 9/ L for HbSS and 330.0 +/- 97.7 X 10 9/ L for HbSC respectively (p= 0.000).

**Table 1:** Mean and standard deviation of coagulation parameters

TEST	HbAA	HbSS	HbSC	Significance
PT (seconds)	12.9 + /- 1.2	13.4 + /- 1.4	13.1 + /- 1.1	F= 1.671, P= 0.192
APTT (seconds)	28.1 + /- 3.8	24.1 + /- 6.6	21.8 + /- 3.8	F= 16.954, P= 0.000
Fibrinogen Concentration(g/L)	2.9 + /- 0.4	1.6 + /- 0.7	3.2 + /- 0.6	F= 87.537, P= 0.000
Platelet Count ( X 10^9^ / L)	245.8 + /- 77.7	445.7 + /- 128.3	330.0 + /-97.7	F= 38.102, P= 0.000

Mean APTT was 28.1 +/- 3.8 seconds in controls while it was 24.1 + /- 66 seconds in HbSS patients and 21.8 +/- 3.8 seconds in HbSC (p = 0.000) The mean concentration of fibrinogen in HbSS was significantly lower at 1.6 +/- 0.7 g/L than HbSC 3.2 +/- 0.6 g/L and controls 2.9 +/- 0.4 g/L (p =0.000) shown in Table 1: mean and standard deviation of coagulation parameters.

## Discussion

The mean platelet count was significantly higher in HbSS and HbSC patients compared to the HbAA controls; this is in keeping with earlier reports of elevated platelet count in SCD [14], which was attributed to the auto-splenectomy and/or loss of splenic function that frequently occurs in HbSS patients.

The lower prevalence of auto-splenectomy and / or loss of splenic function in HbSC patients compared to HbSS patients [15, 16] may explain why their mean PC was lower than that of HbSS patients, though this was higher than the mean counts for HbAA controls. This suggests that though splenic function may be better preserved in HbSC patients than HbSS patients; it was still impaired compared to HbAA controls.

However, the mean platelet count for HbSS and HbSC patients in this study were both higher than that from an earlier study from Benin City, Nigeria [14] where a mean platelet count of 222.41 +/- 40.5X 10^9^/ L was reported for SCD patients compared to 206.2 +/- 62.0X 10^9^/ L for controls, although there was no distinction between the various forms of SCD in the earlier study from Benin City, thus it is possible that their study population included both HbSS, HbSC and other forms of SCD patients.

The mean fibrinogen concentration (FC) in our patients in steady state was within our reference range of 1.5 to 4.0 g/L. This was significantly higher in HbSC than HbSS patients and HbAA controls. However, mean FC in HbSS patients was unexpectedly lower than that of HbAA control subjects. This was in contrast to earlier studies which had reported elevated levels of fibrinogen in HbSS patients in steady state, with a further elevation during crises [14, 17]. The mean FC of 1.6 +/- 0.7 g/ L for HbSS patients found in this study is comparable to a mean concentration of 2.2 +/- 0.3 g/L earlier reported by Buseri et al [18].

The mean APTT was significantly shorter in HbSC patients than both HbSS and HbAA while there was no significant difference in the mean PT among the 3 study groups. The PT was shortest in HbAA controls. It has been previously reported that there is an elevation of Factor VIII in SCD in the absence of significant elevation of other coagulation factors [14, 19], this isolated elevation of Factor VIII concentration may explain the significantly shorter APTT found in the HbSC and HbSS patients we studied. Our reference range for APTT is 26 – 36 seconds.

The mean PT found in this study among HbSC and HbSS patients is similar to results from a study in Sudan where a normal PT was also found [20] this may be due to a reduced concentration of Factor V in SCD patients [14]. This reduction in Factor V concentration may compensate for the expected coagulation activation and shortened PT in SCD [11]. Our reference range for PT is 12 – 16 seconds.

The higher mean fibrinogen concentration found in HbSC may predispose them to elevated plasma viscosity; this along with their shorter APTT may lead to an increased risk of thrombotic events [9].

## Conclusion

Our study demonstrates that a variation exists in PC, APTT and FC between HbSC and HbSS patients as well as between both forms of SCD and HbAA controls. We conclude that the elevated FC and shortened APTT may play a role in the development of some of the complications more characteristic of HbSC such as increased prevalence of retinopathy and osteonecrosis. These results are pointers to the fact that HbSC is not merely a milder form of HbSS; thus, both diseases should be seen as different entities with regards to pathogenesis of the disease, development of complications and approaches for management.
